# Metabolic Brain Network Analysis With ^18^F-FDG PET in a Rat Model of Neuropathic Pain

**DOI:** 10.3389/fneur.2021.566119

**Published:** 2021-07-02

**Authors:** Bei-Bei Huo, Mou-Xiong Zheng, Xu-Yun Hua, Jun Shen, Jia-Jia Wu, Jian-Guang Xu

**Affiliations:** ^1^School of Rehabilitation Science, Shanghai University of Traditional Chinese Medicine, Shanghai, China; ^2^Department of Traumatology and Orthopedics, Yueyang Hospital, Shanghai University of Traditional Chinese Medicine, Shanghai, China; ^3^Department of Orthopedics, Guanghua Hospital of Integrative Chinese and Western Medicine, Shanghai University of Traditional Chinese Medicine, Shanghai, China; ^4^Department of Rehabilitation Medicine, Yueyang Hospital, Shanghai University of Traditional Chinese Medicine, Shanghai, China

**Keywords:** metabolic brain network, ^18^F-FDG, neuropathic pain, PET/CT, rat

## Abstract

Neuropathic pain has been found to be related to profound reorganization in the function and structure of the brain. We previously demonstrated changes in local brain activity and functional/metabolic connectivity among selected brain regions by using neuroimaging methods. The present study further investigated large-scale metabolic brain network changes in 32 Sprague–Dawley rats with right brachial plexus avulsion injury (BPAI). Graph theory was applied in the analysis of 2-deoxy-2-[18F] fluoro-D-glucose (^18^F-FDG) PET images. Inter-subject metabolic networks were constructed by calculating correlation coefficients. Global and nodal network properties were calculated and comparisons between pre- and post-BPAI (7 days) status were conducted. The global network properties (including global efficiency, local efficiency and small-world index) and nodal betweenness centrality did not significantly change for all selected sparsity thresholds following BPAI (*p* > 0.05). As for nodal network properties, both nodal degree and nodal efficiency measures significantly increased in the left caudate putamen, left medial prefrontal cortex, and right caudate putamen (*p* < 0.001). The right entorhinal cortex showed a different nodal degree (*p* < 0.05) but not nodal efficiency. These four regions were selected for seed-based metabolic connectivity analysis. Strengthened connectivity was found among these seeds and distributed brain regions including sensorimotor area, cognitive area, and limbic system, etc. (*p* < 0.05). Our results indicated that the brain had the resilience to compensate for BPAI-induced neuropathic pain. However, the importance of bilateral caudate putamen, left medial prefrontal cortex, and right entorhinal cortex in the network was strengthened, as well as most of their connections with distributed brain regions.

## Introduction

Brachial plexus avulsion injury (BPAI) is a serious peripheral nerve injury causing partial or total loss of motor, sensory, and autonomic functions (1). Neuropathic pain is regarded as one of the most serious and intractable consequences of BPAI that greatly influences patients' quality of life (2–4). According to previous studies, remodeling of cortical and subcortical neural circuitry has been a potentially important mechanism in developing and sustaining neuropathic pain (5–8). Multiple structures have been proved to be involved in this process (9–13).

We have previously revealed changed regional brain activities following BPAI-induced neuropathic pain in rats by ^18^F-FDG PET images (14–16). But growing evidence indicated that reorganization of large-scale brain connectivity and network is also a critical mechanism for chronic pain (17). Studies also showed that chronic pain was related to reorganization over the whole brain and connectivity among distributed brain regions (18–20). The PET image is a direct index of glycometabolism, thus a more reliable measure for brain neuronal activities. It has been widely used to analyze brain metabolism with high sensitivity quantitively (21).

Furthermore, different from functional brain networks based on time series, PET-based metabolic networks are an inter-subject network extracted from a group of subjects (22). Few studies have focused on the topological organization of BPAI of rats based on the metabolic networks. In the present study, graph theory was applied for the inter-subject metabolic network analysis in SD rats with unilateral BPAI. Graph theoretical approaches provided a method for analyzing functional correlations among all the brain regions and the whole brain network efficiency. Thus, it allowed us to explore the entire assembling of the network. Both global and local topologic features were employed to quantify changes following BPAI. We also provided a more global view of information flow in the brain by longitudinally comparing pre- and post-BPAI status which would be inapplicable in human researches.

## Materials and Methods

### Animals

In the present study, 32 adult female SD rats, which weighed 180–200 g and were aged 6–8 weeks, were used. Rats were raised in a laboratory environment without any surgery or examination for at least 1 week. The total rats were obtained from Shanghai SLACK Laboratory Animal Limited Liability Company (Shanghai, China) and they were housed under stable conditions with food and water freely accessible. The environmental temperature was 20–22°C, and the 12/12 h light–dark cycle was stable.

All protocols and procedures of animal use were carried out based on the *Guide for the Care and Use of Laboratory Animals* described by the U.S. National Institutes of Health. All surgical procedures and protocols were approved by the Animal Ethical Committee of Shanghai University of Traditional Chinese Medicine. The number of animals used were minimized.

### Animal Model

The procedure of BPAI has been described in our previous articles (14–16). Briefly, the rats were anesthetized using sodium pentobarbital with an intraperitoneal injection (40 mg/kg), then placed on a thermostatic operating table. A longitudinal incision (~4 cm) was made from the occipital protuberance to scapular angulus superior using surgical scissors. The muscles and fascial layers were identified and retracted with a sterile blunt tweezer. Under an operating microscope (magnification ×10), the right brachial plexus roots were recognized. When the spine was exposed, hemilaminectomies were performed to explore the nerve roots of C5 to T1. They were clearly dissociated and then extracted from the spinal cord. Both dorsal and ventral nerve rootlets were confirmed and avulsed under direct vision. To avoid the infection, penicillin powder was applied for the wounds. All operations were performed by the same experimenter to ensure homogeneity.

### Acquisition of PET Image

Brain PET images of all rats were obtained both pre-BPAI and 1 week post-BPAI, respectively. The rats were fasted overnight before scanning to enhance the absorption of tracer in the brain. In the experiment, 0.5 mCi of ^18^F-FDG tracer was injected via the tail vein. After a 30-min period for adequate uptake, the rats were transiently anesthetized with halothane gas for 1 min before lying on the bed of PET/CT R4 (Siemens Inc., USA). During scanning, the anesthetized rats were stabilized with adhesive tape. The dose of halothane gas was set as an induction dose of 5% and a maintenance dose of 1.5%. Images of rats were reconstructed in OSEM3D mode (128 × 128 matrix) and attenuation corrections were performed. The cross-sectional, coronal, and sagittal images were obtained for further analysis.

### Data Processing and Analysis

All PET data (pre- and post-BPAI images) were processed using the Statistical Parametric Mapping 8 (SPM8) toolbox (http://www.fil.ion.ucl.ac.uk/spm/) which was based on the Matlab R2014a platform (Mathworks, Inc., Natick, MA, USA). Firstly, Image J software (National Institutes of Health, Bethesda, MD, USA) was used to convert the raw images from DICOM format to NIFTI format. Secondly, voxels were upscaled with a 10× scale factor to be adequate for the algorithm implemented in SPM8 and re-sliced to 2 × 2 × 2 mm^3^. The individual image was normalized to a standard rat template following the study by Schwarz et al. (23). Finally, data were smoothed with a full width at half maximum (FWHM = 4 mm) of twice as the voxel size. The mean ^18^F-FDG uptake in each region was normalized by the mean ^18^F-FDG uptake of whole brain to correct for variability in injected activity (21, 24).

### Metabolic Brain Network Construction

In each rat, brain volumes were segmented into 96 regions using the rat brain atlas reported by Schwarz et al. (23) (Supplementary Table 1). All the brain regions were defined as nodes of the network (96 nodes in total). The standard uptake value (SUV) of ^18^F-FDG of regions of interest (ROIs) were extracted for analysis. Inter-nodal Pearson's correlation coefficients between every two brain regions were calculated.

A weighted, undirected inter-subject network composed of 4,608 edges was constructed for the pre- and post-BPAI rats, respectively. Sparsity was defined as the ratio between the number of existing edges and all possible edges in a network. It was used to transfer an unweighted matrix to a binary one. A series of sparsity thresholds ranging from 10 to 50% with an increment of 1% were applied to compare network properties under different thresholds. The network construction process was performed with the Matlab R2014a software.

### Measures of Global Network Property

Small-world measures of a network including clustering coefficient (*C*_*p*_) and path length (*L*_*p*_) were initially estimated. Clustering coefficient *C*_*i*_ of a node *i* was defined as the ratio of the number of existing connections among the nodes' neighbors and all their possible connections. *C*_*p*_ of a network was the average of the clustering coefficients over all nodes. It quantified the local interconnectivity of the network. *L*_*p*_ of a network was the shortest path length (number of edges) required to transfer from one node to another averaged over all pairs of nodes. It represented the overall routing efficiency of a network.

To estimate the small-world properties, *C*_*p*_ and *L*_*p*_that were driven from the networks were further scaled with the mean of *C*_*p*−*s*_ and *L*_*p*_ of 100 random networks (γ = *C*_*p*_/*C*_*p*−*s*_, λ = *L*_*p*_/*L*_*p*−*s*_, and small-worldness scalar σ = γ/λ). In a typical small-world network, γ > 1 and λ ≈ 1, and σ should be more than 1 (25).

Network efficiency, including global efficiency (*E*_*global*_) and local efficiency (*E*_*local*_), was also estimated. For a network *G* with *N* nodes and *K* edges,

$$\begin{array}{l} E_{global}=\;\frac{1}{N\left(N-1\right)}\underset{i\neq j\in G}{\sum }\frac{1}{l_{ij}},\\ E_{local}=\frac{1}{N}\underset{i\in G}{\sum }E_{global}\left(G_{i}\right), \end{array}$$

where *l*_*ij*_ is the shortest path length between node *i* and node *j* in *G*, *E*_*global*_(*G*_*i*_) is the global efficiency of *G*_*i*_, the subgraph composed of the neighbors of node *i*. Global efficiency measures the ability of parallel information transmission over the network, while local efficiency measures the fault tolerance of the network.

### Measures of Nodal Network Properties

Three measures of nodal network properties in each node were employed to investigate the topological architecture of the metabolic brain networks: degree, betweenness centrality, and efficiency (26).

Degree is the number of connections linked to a given node, which in practice is also equal to the number of neighbors of the node. It is a measure of connectivity of a node with the rest of the nodes in a network (26).

In a network *G* with *N* nodes and *K* edges, the degree *k*_*i*_ of a node *i* is defined as:

$$\begin{array}{l} k_{i}=\;\underset{j\in G}{\sum }\alpha_{ij}. \end{array}$$

where α_*ij*_ is the *i*th row and *j*th column element of the adjacency matrix *A*.

Betweenness centrality is defined as the fraction of all shortest paths in the network that passthrough a given node. It describes the influence of an index node over information flow in a network. Bridging nodes that connect disparate parts of the network usually have a high betweenness centrality (27).

Betweenness centrality of node *i*,

$$\begin{array}{l} b_{i}=\;\frac{1}{\left(n-1\right)\left(n-2\right)}\underset{\begin{array}{c} h,j\epsilon N\\ h\neq j,h\neq i,j\neq i \end{array}}{\sum }\frac{\rho_{hj}\left(i\right)}{\rho_{hj}}, \end{array}$$

where ρ_*hj*_is the number of shortest paths between *h* and *j*, and ρ_*hj*_(*i*) is the number of shortest paths between *h* and *j* passing through *i*.

Nodal efficiency is the inverse of the harmonic mean of the shortest path length between a given node and all other nodes. It reveals the capability of an index node to propagate and exchange information with all other nodes in a network (28). The nodal efficiency of node *i* is measured as:

$$\begin{array}{l} e_{i}=\;\frac{1}{N-1}\underset{j\neq i\in G}{\sum }\frac{1}{l_{ij}}\;, \end{array}$$

where *l*_*ij*_ is the shortest path between node *i* and node *j*.

### Statistical Analysis

After the image preprocessing, permutation testing (*n* = 10,000) was performed to estimate the significance for the correlation coefficients of SUV between every two brain regions among all the rats. In each permutation, all pre- and post-BPAI images were randomly reassigned into two groups. The false discovery rate (FDR) method was used for correction of multiple comparisons in the network properties, and the significance level was set at *p* = 0.05.

Seed-based analysis was then performed. Correlation coefficients between the extracted inter-subject SUV values and all the rest of the nodes of the brain were calculated. The differences between pre- and post-BPAI status were compared. Permutation testing (*n* = 10,000) was also performed to evaluate the significance, and the significance level was set at *p* = 0.05.

## Results

### Animals

All rats showed normal state and flexible activity before BPAI. The right forelimb was paralyzed entirely and nonresponsive to any stimulation following BPAI. Within the 3 days post-BPAI, the rats showed slight weakness but gradually recovered to pre-operative status. Autotomic behavior of biting the toes on the injured side was observed. Horner's syndrome was also shown, such as concave eyeballs and ptosis. Abnormal foraging activity, infection, and cervical instability was not observed.

### Small-World Properties

**Figure 1:**
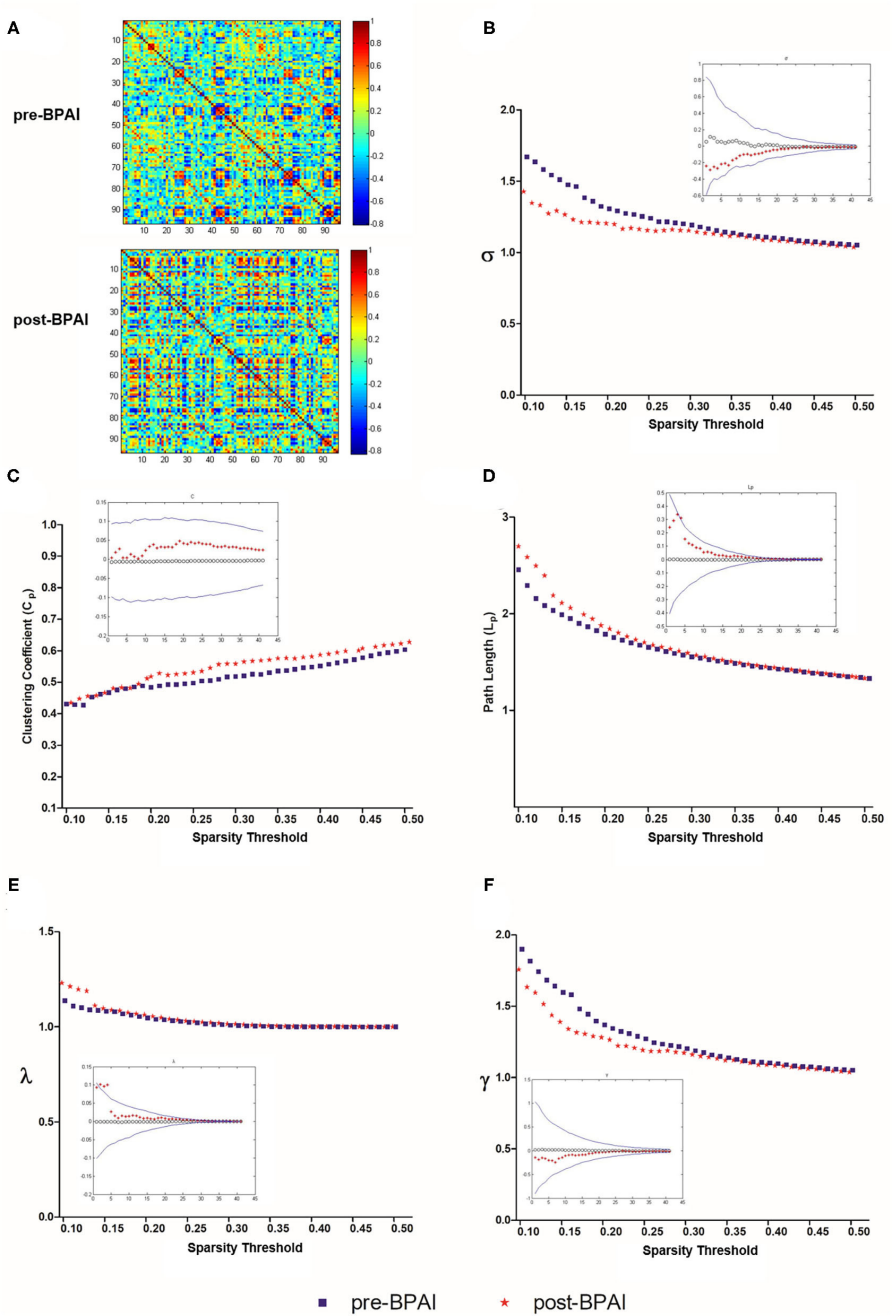
Matrices of brain metabolic connectivities and global network properties [Small-world index σ, clustering coefficient (C_p), normalized clustering coefficient (λ), shortest path length (L_p), and normalized shortest path length (γ)] in pre- and post-BPAI rats. **(A)** Matrices of brain metabolic connectivities across rats based on 96 brain regions using the Schwarz rat brain atlas. The above matrix was derived from rats before the BPAI procedure, while the below one from post-BPAI rats. **(B–F)** Small-world index σ, clustering coefficient (*C*_*p*_), normalized clustering coefficient (λ), shortest path length (*L*_*p*_), and normalized shortest path length (γ) of pre- and post-BPAI rats over different sparsity thresholds ranged from 0.1 to 0.5, with an increment of 0.01. No significant difference was found between pre- and post-BPAI rats under all sparsity thresholds following permutation testing (*n* = 10,000). Blue square plots represent pre-BPAI rats, while red star plots represent post-BPAI rats. BPAI, brachial plexus avulsion injury.

Weighted, undirected correlation matrices of pre- and post-BPAI rats were constructed for comparison (Figure 1A). For the selected range of sparsity thresholds, rats in pre- and post-BPAI status both showed σ >1, indicating rats still possessed small-world property (Figure 1B). However, according to the permutation results, no significant difference was found regarding the clustering coefficient (*C*_*p*_) and path length (*L*_*p*_), as well as λ and γ (*p* > 0.05) (Figures 1C–F).

**Figure 2:**
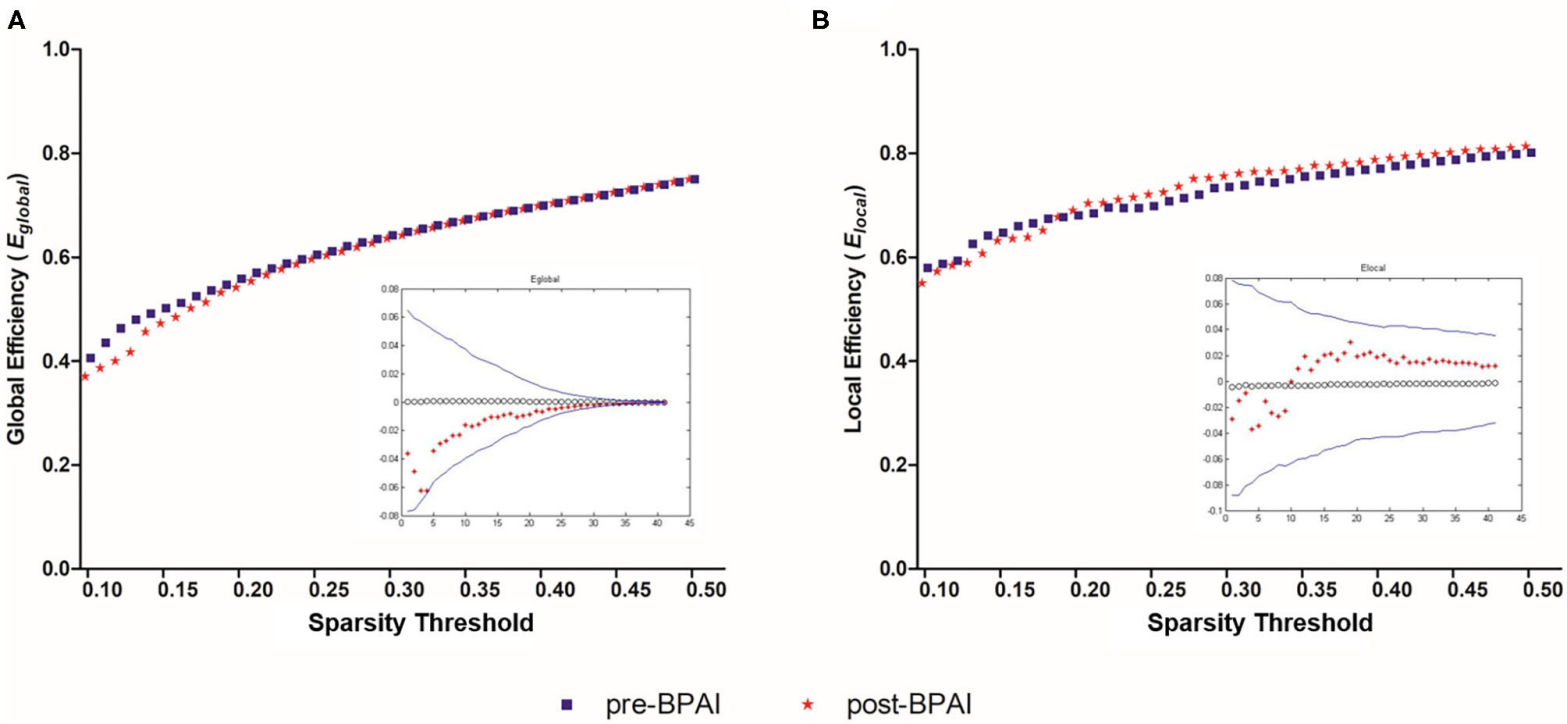
Global and local efficiencies in pre- and post-BPAI rats. Global efficiency **(A)** and local efficiency **(B)** in pre- and post-BPAI rats over different sparsity thresholds ranged from 0.1 to 0.5, with an increment of 0.01. No significant difference was found between pre- and post-BPAI rats under all sparsity thresholds following permutation testing (*n* = 10,000). Blue square plots represent pre-BPAI rats, while red star plots represent post-BPAI rats. BPAI, brachial plexus avulsion injury.

Similarly, no significant difference was noted between pre- and post-BPAI status in either global efficiency (*E*_*global*_) or local efficiency (*E*_*local*_) under any of the selected spasticity thresholds (*p* > 0.05) (Figures 2A,B).

### Nodal Network Properties

Both nodal degree and nodal efficiency measures were different in the left caudate putamen, left medial prefrontal cortex, and right caudate putamen (FDR correction, *p* < 0.05). The right entorhinal cortex showed a different nodal degree (FDR correction, *p* < 0.05) but not nodal efficiency (FDR correction, *p* > 0.05), but no significant difference was found in nodal betweenness centrality in any node (FDR correction, *p* > 0.05). The four brain regions that presented differences in any of the three nodal network properties were shown in Supplementary Table 2.

### Changes of Metabolic Connectivity

**Figure 3:**
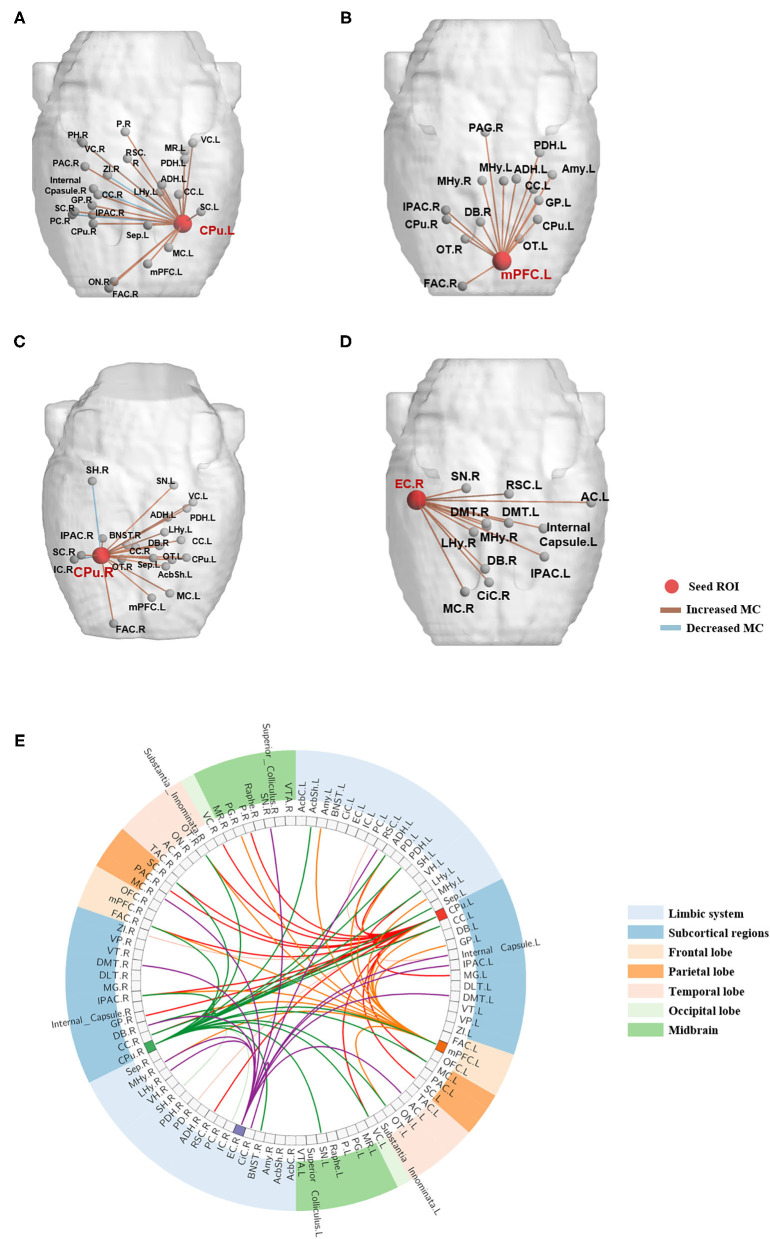
Metabolic connectivities between the seed brain regions and the resting brain significantly changed following BPAI. **(A–D)** Significantly changed metabolic connectivities following BPAI based on seed regions selected from brain network analysis. Left CPu **(A)**, left mPFC **(B)**, right CPu **(C)**, right EC **(D)** were four seed nodes that showed significantly changed nodal properties in rats following BPAI. **(E)** The global view of metabolic connectivities based on seed-based analysis in the BPAI rats. Deep-colored lines sprouted from left CPu, left mPFC, right CPu, and right EC indicated significantly increased metabolic connectivities in rats following BPAI, while light-colored lines indicated significantly decreased metabolic connectivities. BPAI, brachial plexus avulsion injury; other abbreviations were shown in Supplementary Table 1.

The four brain regions that presented changed nodal network properties following BPAI were chosen as seed ROIs. Metabolic connectivity between the seed ROIs and all the rest of the brain regions was calculated, respectively. Changes (mainly increased) could be observed in both intra- and inter-hemisphere metabolic connectivity (Figure 3 and Supplementary Table 3).

Increased metabolic connectivity included the following pairs (five most significantly increased connectivity based on each seed ROIs were listed): (1) left caudate putamen (CPu.L): right frontal association cortex (FAC.R), IPAC (IPAC.R), globus pallidus (GP.R) visual cortex (VC.R), and left lateral hypothalamus (LHy.L), etc.; (2) left medial prefrontal cortex (mPFC.L): right frontal association cortex (FAC.R), medial hypothalamus (MHy.R), diagonal band (DB.R) and left anterodorsal hippocampus (ADH.L) and caudate putamen (CPu.L), etc.; (3) right caudate putamen (CPu.R): right frontal association cortex (FAC.R), bed nucleus of the stria terminals (BNST.R) and left posterodorsal hippocampus (PDH.L), substatia nigra (SN.L) and septum (Sep.L), etc.; (4) right entorhinal cortex (EC.R): right medial hypothalamus (MHy.R), diagonal band (DB.R), motor cortex (MC.R), and left auditory cortex (AC.L) and IPAC (IPAC.L).

Decreased metabolic connectivities only included the following pairs: (1) left caudate putamen (CPu.L): right piriform cortex (PC.R), zona incerta (ZI.R), posterior hippocampus (PH.R) and internal capsule (Internal_Capsule.R); (2) right caudate putamen (CPu.R): right insular cortex (IC.R) and subiculum hippocampus (SH.R).

## Discussion

BPAI-induced neuropathic pain is a specific type of deafferentation pain, the mechanism of which may be distinct from acute pain (29). ^18^F-FDG is a widely used marker for detecting glucose metabolic rates in the brain. It is also regarded as a “golden index” for neuronal activity, thus, it is a valuable measure for investigating pain-related brain activities (30).

Our previous study has revealed a specific pattern of brain metabolic changes following unilateral BPAI. Metabolic connectivity based on sensorimotor-related seed ROIs indicated extensive involvement of bilateral hemispheres in the development and persistence of pain, including motor cortex, somatosensory cortex, dorsolateral thalamus, and caudate putamen (16). Meanwhile, neuropathic pain would also cause plastic changes in interconnected regions instead of segregated regions alone. For example, the concept of the “pain matrix” has been raised to describe a group of brain regions jointly activated by neuropathic pain (31). Brain networks, including sensorimotor, cognitive, emotional regions, may be involved in this procedure, and network analysis has become an important method for measuring the function of the brain under different pathological statuses.

Previous studies mainly focused on the functional connected network by analyzing correlations depending on signal fluctuations with time. For example, Barrière et al. (32) used a spared nerve injury model that caused pain-associated comorbidities, and found that neuropathic pain profoundly altered the intrinsic organization of the brain functional network by using functional MRI (fMRI). Baliki et al. (33) revealed intrinsic brain reorganization by resting-state fMRI functional connectivity-based whole-brain network properties in rats receiving spared nerve injury (SNI). But very few studies have referred to longitudinal changes of metabolic brain network associated with BPAI. Metabolic network was thus a superior measure for revealing interaction between related brain regions. Kim et al. (18) analyzed FDG micro-PET images in awake rats with ligations on the spinal nerve. They found that small-worldness decreased and the network was separated into fragmented modular structures. To our knowledge, the present study firstly described small-world properties in the brain metabolic network by PET in animal models of BPAI-induced neuropathic pain.

As previous research has indicated, the human brain is a typical small-world network (34–36). Our research showed that metabolic networks in rats both pre- and post-BPAI status also possessed small-world characteristics. However, we did not note significantly the changed small-world index, local and global efficiencies for all the defined sparsity thresholds. A small-world network has the features of both a random network and a regular network. Both global and local efficiency tend to be high in a small-world. The present results reflected that rats' small-world properties were not significantly disturbed following BPAI, indicating that the information exchange efficiency of the whole brain network would not decline. The present results were similar to Baliki et al.'s (33) research comparing rats receiving spared nerve injury (SNI) and sham injury. They found that the topological properties of rat brain showed small-world features and there was no difference between SNI and sham groups. The changes of functional connection were localized mainly to the limbic system, as well as between the limbic and nociceptive systems.

Nodal network properties were found changed in bilateral caudate putamens (CPus), left medial prefrontal cortex (mPFC), and right entorhinal cortex (EC). Caudate putamen (i.e., dorsal striatum), which receives signals from the cortex and thalamus, is the gateway to the basal ganglia. It forms the origin of direct-indirect pathways, which are distinct basal ganglia circuits involved in motor control (37). The dorsal striatum handles the input -output of many functions, for example, motor activity (38). The plasticity of the striatum might be related to adaptive motor control and procedural memory (37). The medial prefrontal cortex is important not only in executive function, but also in pain management. The latter relies on its association with the neocortex of the brain, the hippocampus, and regions of the basal nucleus (39). In a rat, the medial prefrontal cortex is also related to working memory, attention, response initiation, and autonomic control and emotion (40–43). The entorhinal cortex is an essential structure for memory formation and consolidation (44, 45). Nodal network properties, including nodal degree and/or nodal efficiency, changed in these regions demonstrated increased importance in the network information exchange. These regions were generally involved in the activities of motor control, emotional behavior, and memory formation. We considered it to be the result of sensorimotor deficits and neuropathic pain-induced cognitive and emotional compensation.

Analysis of seed-based metabolic connectivity further indicated that brain regions potentially changed information exchange efficiency with these four regions following BPAI. The increased metabolic connectivity with bilateral caudate putamens (CPus) mostly involved sensorimotor information processing-integration, cognitive function and limbic system. The left medial prefrontal cortex (mPFC.L) most likely increased its connectivity with regions responsible for sensory information integration-transfer, learning and memory, and limbic system. The right entorhinal cortex (EC) showed increased connectivity with regions of autonomic system modulation, and the limbic and sensorimotor systems. Decreased connectivities were much lower and included regions of olfactory sensation, memory, and social emotion. The results indicated that distributed brain regions changed metabolic connectivity with these seed regions.

In summary, we applied the specific model of unilateral BPAI to induce neuropathic pain used in our previous researches. The properties of metabolic brain network in pre- and post-BPAI conditions were compared using ^18^F-FDG PET imaging in SD rats. We found that both pre- and post-BPAI rats possessed small-world properties. But the small-world index, global efficiency, and local efficiency did not significantly change following BPAI. Nodal betweenness centrality did not change either, but the nodal degree and nodal efficiency increased in the bilateral caudate putamen, left medial prefrontal cortex, and/or right entorhinal cortex, showing increased importance of these regions in the network. Their metabolic connections with many regions also changed (mainly strengthened). Therefore, the metabolic brain network has the resilience following BPAI-induced neuropathic pain. Increased efficiency in regional nodes might be an important compensatory mechanism.

### Limitations

A potential limitation was that we assessed short-term changes in metabolic connectivity in anesthetized animals. A long-term evaluation in awake rats that involves more longitudinal time points and additional intervention would be worthwhile in the future. Meanwhile, the present results in rats may not be wholly extrapolated to humans as human brains are more functionally and structurally complicated. It is still necessary to conduct further investigations in clinical patients. Comparisons between BPAI patients and healthy subjects would provide helpful information as well.

## Data Availability Statement

The original contributions presented in the study are included in the article/Supplementary Material, further inquiries can be directed to the corresponding author/s.

## Ethics Statement

The animal study was reviewed and approved by Animal Ethical Committee of Shanghai University of Traditional Chinese Medicine.

## Author Contributions

J-GX, X-YH, and B-BH: conception and study design. B-BH and JS: acquisition of PET/CT data. X-YH, B-BH, and J-JW: analysis and interpretation of PET/CT data. B-BH and M-XZ: drafting the article. J-GX: approval of the final version to be published. All authors contributed to the article and approved the submitted version.

## Conflict of Interest

The authors declare that the research was conducted in the absence of any commercial or financial relationships that could be construed as a potential conflict of interest.

## Abbreviations

^18^F-FDG: 2-deoxy-2-[18F] fluoro-D-glucose
BPAI: Brachial plexus avulsion injury
C_p_: Clustering coefficient
L_p_: Shortest path length
E_global_: Global efficiency
E_local_: Local efficiency.
