# Supplementary material for: Metabolic Brain Network Analysis With 18F-FDG PET in a Rat Model of Neuropathic Pain
Source: Front Neurol. 2021 Jul 2;12:566119. doi: 10.3389/fneur.2021.566119 (PMC8284720; doi:10.3389/fneur.2021.566119)
Supplement: Supplementary file 1 [file Data_Sheet_1.PDF]

Supplemental table 1 Anatomical brain regions in the rat brain atlas and corresponding abbreviations

No.	Left Hemisphere	No.	Right Hemisphere
1	L_Nucleus Accumbens—Core (Acbc.L)	49	R_Nucleus Accumbens—Core (Acbc.R)
2	L_Nucleus Accumbens—Shell (AcbSh.L)	50	R_Nucleus Accumbens—Shell (AcbSh.R)
3	L_Amygdala (Amy.L)	51	R_Amygdala (Amy.R)
4	L_Bed Nucleus of the Stria Terminalis (BNST.L)	52	R_Bed Nucleus of the Stria Terminalis (BNST.R)
5	L_Caudate Putamen (CPu.L)	53	R_Caudate Putamen (CPu.R)
6	L_Corpus Collosum (CC.L)	54	R_Corpus Collosum (CC.R)
7	L_Cortex—Auditory (AC.L)	55	R_Cortex—Auditory (AC.R)
8	L_Cortex—Cingulate (CiC.L)	56	R_Cortex—Cingulate (CiC.R)
9	L_Cortex—Entorhinal (EC.L)	57	R_Cortex—Entorhinal (EC.R)
10	L_Cortex—Frontal Association (FAC.L)	58	R_Cortex—Frontal Association (FAC.R)
11	L_Cortex—Insular (IC.L)	59	R_Cortex—Insular (IC.R)
12	L_Cortex—Medial Prefrontal (mPFC.L)	60	R_Cortex—Medial Prefrontal (mPFC.R)
13	L_Cortex—Motor (MC.L)	61	R_Cortex—Motor (MC.R)
14	L_Cortex—Orbitofrontal (OFC.L)	62	R_Cortex—Orbitofrontal (OFC.R)
15	L_Cortex—Parietal Association (PAC.L)	63	R_Cortex—Parietal Association (PAC.R)
16	L_Cortex—Piriform (PC.L)	64	R_Cortex—Piriform (PC.R)
17	L_Cortex—Retrosplenial (RSC.L)	65	R_Cortex—Retrosplenial (RSC.R)
18	L_Cortex—Somatosensory (SC.L)	66	R_Cortex—Somatosensory (SC.R)
19	L_Cortex—Temporal Association (TAC.L)	67	R_Cortex—Temporal Association (TAC.R)
20	L_Cortex—Visual (VC.L)	68	R_Cortex—Visual (VC.R)
21	L_Diagonal Band (DB.L)	69	R_Diagonal Band (DB.R)
22	L_Globus Pallidus (GP.L)	70	R_Globus Pallidus (GP.R)
23	L_Hippocampus—Anterodorsal (ADH.L)	71	R_Hippocampus—Anterodorsal (ADH.R)
24	L_Hippocampus—Posterior (PH.L)	72	R_Hippocampus—Posterior (PH.R)
25	L_Hippocampus—Posterodorsal (PDH.L)	73	R_Hippocampus—Posterodorsal (PDH.R)
26	L_Hippocampus—Subiculum (SH.L)	74	R_Hippocampus—Subiculum (SH,R)
27	L_Hippocampus—Ventral (VH.L)	75	R_Hippocampus—Ventral (VH.R)
28	L_Hypothalamus—Lateral (LHy.L)	76	R_Hypothalamus—Lateral (LHy.R)
29	L_Hypothalamus—Medial (MHy.L)	77	R_Hypothalamus—Medial (MHy.R)
30	L_Internal Capsule (Internal_Capsule.L)	78	R_Internal Capsule (Internal_Capsule.R)
31	L_IPAC (IPAC.L)	79	R_IPAC (IPAC.R)
32	L_Medial Geniculate (MR.L)	80	R_Medial Geniculate (MR.R)
33	L_Mesencephalic Region (MR.L)	81	R_Mesencephalic Region (MR.R)
34	L_Olfactory Nuclei (ON.L)	82	R_Olfactory Nuclei (ON.R)
35	L_Olfactory Tubercle (OT.L)	83	R_Olfactory Tubercle (OT.R)

36	L_Periaqueductal Gray (PG.L)	84	R_Periaqueductal Gray (PG.R)
37	L_Pons (P.L)	85	R_Pons (P.R)
38	L_Raphe (Raphe.L)	86	R_Raphe (Raphe.R)
39	L_Septum (Sep.L)	87	R_Septum (Sep.R)
40	L_Substantia Innominata (Substantia_Innominata.L)	88	R_Substantia Innominata (Substantia_Innominata.R)
41	L_Substantia Nigra (SN.L)	89	R_Substantia Nigra (SN.R)
42	L_Superior Colliculus (Superior Colliculus.L)	90	R_Superior Colliculus (Superior Colliculus.R)
43	L_Thalamus—Dorsolateral (DLT.L)	91	R_Thalamus—Dorsolateral (DLT.R)
44	L_Thalamus—Midline Dorsal (MDT.L)	92	R_Thalamus—Midline Dorsal (MDT.R)
45	L_Thalamus—Ventromedial (VT.L)	93	R_Thalamus—Ventromedial (VT.R)
46	L_Ventral Pallidum (VP.L)	94	R_Ventral Pallidum (VP.R)
47	L_Ventral Tegmental Area (VTA.L)	95	R_Ventral Tegmental Area (VTA.R)
48	L_Zona Incerta (ZI.L)	96	R_Zona Incerta (ZI.R)

Supplemental table 2 Brain regions present significant difference between pre- and post-BPAI rats in nodal network properties

Brain Regions	Nodal Degree			Nodal Efficiency		
	pre-BPAI	post-BPAI	*p*-value	pre-BPAI	post-BPAI	*p*-value
Left Caudate Putamen	5.415	16.660	0.0001*	0.2125	0.2808	0.001*
Left Medial Prefrontal Cortex	6.375	18.770	0.0001*	0.2143	0.2913	0.001*
Right Caudate Putamen	5.895	17.600	0.001*	0.2149	0.2865	0.001*
Right Entorhinal Cortex	3.570	18.295	0.002*	0.1997	0.2900	0.008

BPAI: brachial plexus avulsion injury.
**Significant difference between pre- and post- BPAI rats (false discovery rate (FDR) correction).*

Supplemental table 3 Significantly changed metabolic connectivity between pre- and post-BPAI rats based on seed ROIs that showing changed nodal network properties following BPAI

Seed ROI	Brain Regions of Changed Metabolic Connectivity	Metabolic Connectivity			p-value
		pre-BPAI	post-BPAI	D-value	
CPu.L	**FAC.R**	**0.01**	**0.67**	**0.66**	**0.001**
	**IPAC.R**	**0.05**	**0.64**	**0.59**	**0.011**
	**GP.R**	**0.03**	**0.58**	**0.55**	**0.008**
	**LHy.L**	**0.09**	**0.64**	**0.54**	**0.042**
	**VC.R**	**0.03**	**0.58**	**0.54**	**0.019**
	CC.R	0.28	0.80	0.52	0.013
	Sep.L	0.11	0.62	0.51	0.047
	PDH.L	0.10	0.60	0.50	0.034
	mPFC.L	0.25	0.72	0.47	0.012
	PAC.R	0.10	0.52	0.42	0.015
	P.R	0.19	0.61	0.42	0.044
	VC.L	0.10	0.51	0.40	0.026
	CC.L	0.47	0.87	0.40	0.021
	RSC.R	0.02	0.39	0.37	0.045
	SC.R	0.39	0.73	0.35	0.030
	MC.L	0.40	0.74	0.34	0.016
	CPu.R	0.59	0.90	0.31	0.011
	SC.L	0.36	0.66	0.30	0.033
	ON.R	0.16	0.42	0.26	0.050
	MR.L	0.04	0.26	0.22	0.049
	ADH.L	0.16	0.36	0.20	0.016
	PC.R	*0.46*	*0.02*	*-0.44*	*↓0.006*
	ZI.R	*0.37*	*0.08*	*-0.30*	*↓0.007*
	PH.R	*0.23*	*0.08*	*-0.15*	*↓0.013*
	Internal_Capsule.R	*0.16*	*0.01*	*-0.14*	*↓0.013*
mPFC.L	**FAC.R**	**0.01**	**0.55**	**0.55**	**0.025**
	**Mhy.R**	**0.11**	**0.66**	**0.54**	**0.007**
	**ADH.L**	**0.00**	**0.51**	**0.51**	**0.001**
	**DB.R**	**0.15**	**0.62**	**0.47**	**0.027**
	**Cpu.L**	**0.25**	**0.72**	**0.47**	**0.012**
	OT.R	0.08	0.54	0.46	0.004
	PDH.L	0.28	0.73	0.45	0.021
	CC.L	0.37	0.82	0.45	0.012
	GP.L	0.06	0.49	0.43	0.013
	CPu.R	0.23	0.66	0.43	0.025
	IPAC.R	0.09	0.52	0.42	0.026
	Amy.L	0.06	0.46	0.41	0.027
	OT.L	0.12	0.48	0.36	0.043
	MHy.L	0.29	0.61	0.32	0.048
	PAG.R	0.05	0.30	0.25	0.012
CPu.R	**FAC.R**	**0.08**	**0.72**	**0.64**	**0.001**
	**PDH.L**	**0.04**	**0.66**	**0.63**	**0.003**
	**BNST.R**	**0.06**	**0.67**	**0.61**	**0.001**
	**SN.L**	**0.10**	**0.68**	**0.57**	**0.016**

	**Sep.L**	**0.10**	**0.66**	**0.57**	**0.006**
	CC.L	0.26	0.79	0.53	0.015
	IPAC.R	0.03	0.56	0.52	0.041
	LHy.L	0.25	0.77	0.52	0.012
	DB.R	0.06	0.53	0.47	0.050
	VC.L	0.02	0.47	0.45	0.036
	CC.R	0.43	0.87	0.43	0.011
	mPFC.L	0.23	0.66	0.43	0.025
	MC.L	0.29	0.69	0.40	0.046
	SC.R	0.48	0.81	0.33	0.019
	CPu.L	0.59	0.90	0.31	0.011
	OT.L	0.07	0.34	0.27	0.031
	OT.R	0.20	0.48	0.27	0.029
	AcbSh.L	0.02	0.28	0.27	0.010
	ADH.L	0.03	0.25	0.23	0.006
	IC.R	*0.41*	*0.21*	*-0.20*	*↓0.021*
	SH.R	*0.18*	*0.01*	*-0.17*	*↓0.013*
EC.R	**MHy.R**	**0.06**	**0.85**	**0.78**	**0.015**
	**DB.R**	**0.06**	**0.78**	**0.72**	**0.032**
	**MC.R**	**0.06**	**0.67**	**0.61**	**0.014**
	**AC.L**	**0.07**	**0.62**	**0.55**	**0.041**
	**IPAC.L**	**0.01**	**0.54**	**0.53**	**0.006**
	LHy.R	0.00	0.53	0.52	0.036
	RSC.L	0.04	0.53	0.49	0.033
	CiC.R	0.07	0.49	0.42	0.033
	DMT.R	0.09	0.46	0.38	0.004
	Internal_Capsule.L	0.07	0.30	0.23	0.027
	DMT.L	0.10	0.28	0.18	0.032
	SN.R	0.11	0.26	0.14	0.044

BPAI: brachial plexus avulsion injury; ROI: regions of interest; L: left; R: right.
↓ indicated decreased metabolic connectivity between seed ROI and the brain region following BPAI; while the rest p-values represented increased metabolic connectivity between seed ROI and the brain region following BPAI.
